# Effect of Sample Storage Conditions on Measurements of Salivary Cotinine Levels

**DOI:** 10.3390/metabo10090365

**Published:** 2020-09-08

**Authors:** Fábio Renato Manzolli Leite, Vibeke Baelum, Julie Becker Pajaniaye, Lisbeth Ann Abildtrup, Rodrigo López

**Affiliations:** 1 Section of Periodontology, Department of Dentistry and Oral Health, Aarhus University, Vennelyst Boulevard 9, 8000 Aarhus C, Denmark; jp@dent.au.dk (J.B.P.); rlopez@dent.au.dk (R.L.); 2 Section of Oral Epidemiology and Public Health, Department of Dentistry and Oral Health, Aarhus University, Vennelyst Boulevard 9, 8000 Aarhus C, Denmark; baelum@dent.au.dk; 3 Department of Dentistry and Oral Health, Aarhus University, Vennelyst Boulevard 9, 8000 Aarhus C, Denmark; laa@dent.au.dk

**Keywords:** cotinine, biological markers, tobacco, saliva, metabolites, smokers

## Abstract

Information on smoking exposure obtained with self-reports may be inaccurate. Cotinine has a large half-life and its salivary levels correlate well with plasmatic levels. The influence of storage conditions on the validity and precision of salivary cotinine assessments has rarely been evaluated. Here, smokers donated saliva samples, which were sent for immediate analysis, mail posting, storage at 4 °C for 30 or 90 days, or storage at −20 °C for 30 or 90 days. Cotinine levels were determined using enzyme-linked immune-sorbent assay. Agreement of cotinine level measurements was assessed using Bland-Altman analyses. Average age (years), duration of smoking (years) and number of cigarettes smoked (/day) were 55.4 (±SD 9.4), 35.1 (±SD 11.3), and 15.3 (±SD 7.6). The mean immediate cotinine level was 457 ng/mL (range 11.3 to 1318 ng/mL). Mean cotinine levels in samples analyzed after delay ranged between 433 ng/mL (−20 °C 30 days) and 468 ng/mL (4 °C 30 days). A dose-response gradient was observed in the relationship between salivary cotinine level and self-reported smoking status. A good agreement between cotinine levels for all storage conditions compared with immediate analysis was observed, with average differences ranging from −11 to 24 ng/mL. Cotinine levels remained stable regardless of the tested condition. The stability of salivary cotinine may enable samples to be obtained in difficult-to-reach areas, reduce study costs, and improve the validity of the information on exposure to smoking.

## 1. Introduction

The assessment of exposure to tobacco smoking and nicotine may be required in different settings, such as sports, as well as for clinical and research purposes. In sports, nicotine may have an ergogenic effect and benefit the physical performance of athletes [1]. In the last decade, an increase in the use of nicotine among athletes has been observed, which resulted in nicotine being included in 2012 on the World Anti-Doping Agency Monitoring Program [2]. In the clinical setting, assessment of exposure to tobacco and nicotine may be necessary for legal reasons and for monitoring purposes in smoking cessation programs [3,4,5]. In research, valid and reliable information on smoking and nicotine is needed to evaluate the level of exposure to tobacco or for the purpose of controlling for confounding in statistical analyses of other exposures that might be related to smoking.

Information on smoking and nicotine exposure is frequently sought through self-reports, but the data obtained may be quite inaccurate due to information bias and frequently reflects an underestimation of current exposure [6]. People may be different in their ability and willingness to report correctly their smoking status and nicotine consumption, just as the respondent may be ignorant of the contents of cigarettes and their substitutes. There may also be real variation in the amount of smoke inhaled, which may be especially true for passive smokers [7,8,9]. Consequently, noninvasive and valid methods for the assessment of the exposure to smoking have been developed. Around 85% of nicotine is metabolized into cotinine, which has a half-life of approximately 19 h compared with 2 h for nicotine [10]. Cotinine levels in saliva strongly correlate with those of plasma, and determination of salivary cotinine is cost-effective, non-invasive and requires small sample volumes [11].

The possibility of mailing samples from multiple and/or far away locations to a main laboratory and of keeping samples stored at 4 °C until a reasonably large number of samples have been collected is more convenient and cost-effective than arranging with local partners for immediate analysis or transportation of frozen samples. Even so, very little is known about how sample storage conditions might influence the validity of the assessment of the exposure to tobacco and nicotine. The few studies on the topic have only explored the stability of nicotine or cotinine in samples mailed via United States Postal Service at room temperature, corresponding to an average storage time of 3 to 5 days [6,12,13]. Similarly, little is known on how imprecision in the estimation of salivary cotinine levels may influence our ability to distinguish between real cotinine level changes resulting from reduced smoking and apparent changes resulting from the existence of measurement errors in the assessment of salivary cotinine levels. In the present study, we therefore analyzed the influence of several storage temperatures and storage time on the levels of salivary cotinine, just as we sought to determine the level of salivary cotinine level change that could reliably be considered indicative of a real change.

## 2. Results

The mean age of the 182 participants who provided saliva samples for cotinine level analysis was 55.4 (±SD 9.4) years and the group included 52.3% women. The average smoking duration was 35.1 (±SD 11.3) years and an average of 15.3 (±SD 7.6) cigarettes were reportedly smoked per day. The samples that were mail posted arrived back to the laboratory between 5 and 7 days after posting. Among the 172 participants providing self-reported smoking information, 30 (17%) were classified as Light smokers, 70 persons (41%) were Moderate smokers, and 72 persons (42%) were classified as Heavy smokers.

The 182 samples analyzed immediately showed a mean cotinine level of 457 ng/mL (Table 1) with the individual recordings ranging from 11.3 ng/mL to 1318 ng/mL. Depending on storage condition, the mean cotinine levels ranged between 433 ng/mL (for storage at −20 °C for 30 days) and 468 ng/mL (storage at 4 °C for 30 days).

The mean cotinine level in samples analyzed immediately after thawing showed a clear gradient according to participants’ self-reported smoking status, such that Light smokers had the lowest mean salivary cotinine level (346.4 ng/mL) while Heavy smokers had an average salivary cotinine level of 542.0 ng/mL (Table 2).

**Figure 1:**
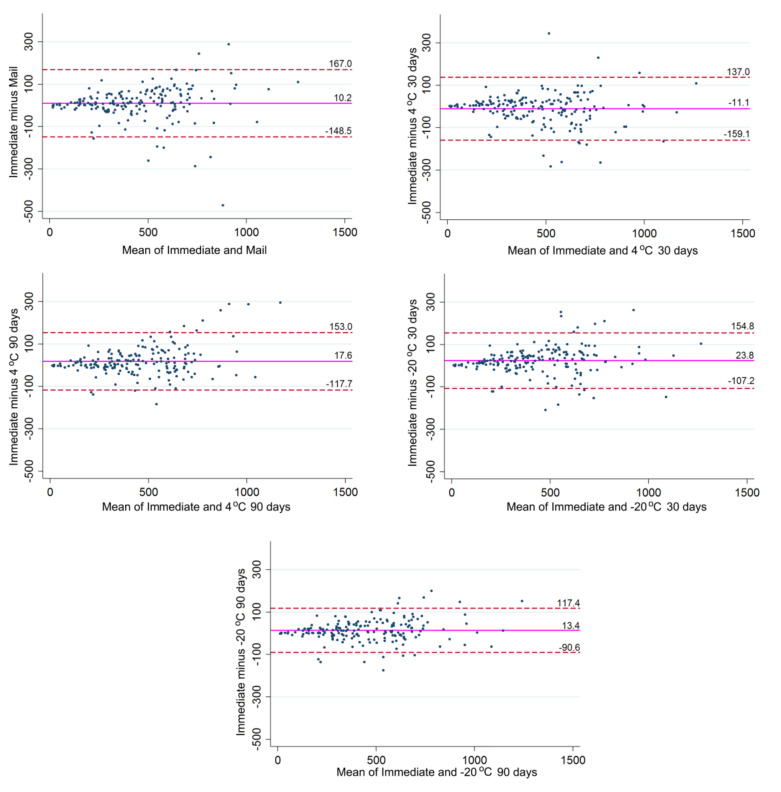
Bland-Altman plots of the five different storage conditions plotted against immediate analysis after −80 °C thawing. The magenta reference line illustrates the mean disagreement; the red dashed lines state the upper and lower limits of agreement. All values are expressed in nanograms of cotinine per milliliter of sample (ng/mL).

Bland-Altman analysis (Figure 1) showed an overall good agreement between the salivary cotinine levels recorded for all storage conditions and those resulting from the immediate analysis after thawing. The average differences between recordings made after storage conditions and those resulting from immediate analysis were generally small, ranging from −11 ng/mL for the difference between immediate analysis and analysis after storage at 4 °C for 30 days to 24 ng/mL for the difference between immediate analysis and analysis after storage at −20 °C for 30 days (Figure 1, Table S1). The limits of agreement, i.e., the interval in which 95% of the differences were observed, were in the order of magnitude of 104–157 ng/mL, depending on the storage condition with which the immediate results were compared (Table S1).

**Figure 2:**
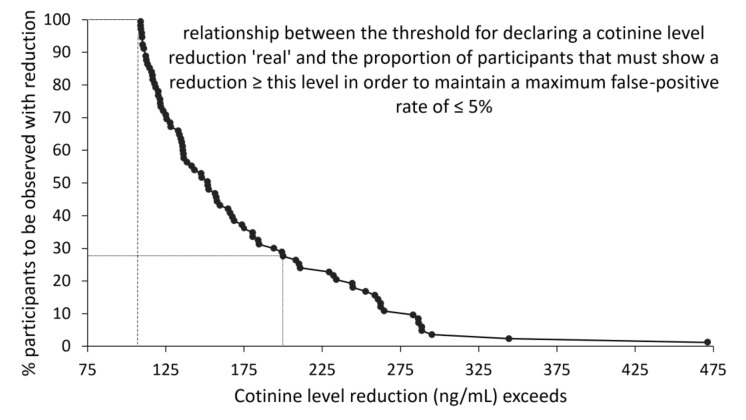
Determination of diagnostic threshold level for a maximum false-positive rate of 5% in a hypothetical smoking cessation study based on cotinine level reduction. The curve shows that only when all participants (100%) in such a study show a reduction in cotinine levels in excess of 108 ng/mL will the false-positive rate be 5% or less (dashed line). Similarly, if 28% or more of the participants show a reduction of 200 ng/mL or more, the false-positive rate will still be 5% or less (dotted line).

One important effect of the presence of measurement error in an assay is that it may become difficult to distinguish real change from measurement error. The problem is not major when groups are contrasted, as one would ordinarily rely on a comparison of the mean values for each group, and use of mean values tend to cancel out any unsystematic errors in the measurements. However, when individual changes are of interest, such as in a study of the success of a smoking cessation intervention, the need arises to distinguish real change from measurement error. As shown in Figure 2, the level of measurement errors observed in the present study would allow for detection with a false-positive rate ≤5%, a cotinine level reduction of 108 ng/mL or more. However, at the lowest cotinine level reduction (108 ng/mL), almost all participants in the hypothetical smoking cessation study would have to display such reductions. The graph also shows that the larger the reduction the fewer the study participants are needed to display such reductions to maintain a low diagnostic false-positive rate. Hence, for cotinine reductions in excess of 200 ng/mL 28% of the study group should show such reductions to maintain the 5% maximum false-positive rate.

## 3. Discussion

In this study, we compared the recordings of cotinine levels observed in aliquots from the same saliva sample stored under different conditions, and found that cotinine levels remained stable regardless of the tested condition. To the best of the authors’ knowledge, no previous studies have tested simultaneously the effect of temperature and time of storage on salivary levels of cotinine. A few studies from the United States can be used to glean information about the stability of cotinine recordings subject to a delayed analysis. Hence, in two studies [12,13] immediate analyses were compared to analyses conducted after samples had been in the mail system (no cooling) for a mean time of 3 to 5 days, and they reported relatively stable cotinine levels. In a third study [6], three of the nineteen valid saliva samples were inadvertently exposed to room temperature for twenty days before analyses, and although the small sample size precludes valid conclusions, there was no indication of a decline in the cotinine level. Taken together, these and the present results indicate that saliva samples obtained under field conditions may be transported to a site of collection and stored under easily attainable cooling conditions for up to three months without jeopardizing the validity of the information on tobacco exposure recorded from salivary cotinine measurements, thus facilitating cost-effective assessments.

To the best of our knowledge, no assay reproducibility data have been published over and beyond those stated in the package insert of the Salimetrics assay. According to the insert, the kit was validated by Salimetrics using different known quantities of an endogenous cotinine with ≥94.0% recovery levels. The inter-assay reproducibility, expressed as the coefficient of variation (CV) based on the estimation of ‘the mean of average duplicates for eight separate runs’, is 4.2% at a concentration ~100 ng/mL and 9.0% at ~6 ng/mL. In this study, the average CV for duplicate readings of experimental samples was 2.36% (standard error mean 0.33%), while the average CV of quality control samples was 3.64% (standard error mean 1.03%). Since the standard error percentage, contrary to CV, reduces with increasing numbers of replicates, the standard error percentage provides a better and more precise estimate [14]. It is important to highlight that the Food and Drug Administration determined CV as a standard measure and inter-assay CV values are acceptable if ≤15% [15]. While these estimates validate the assay, they do not answer the question of how different single assessments of the same sample may be, and how this difference might vary over the range for the assay, which is 0.8–200 ng/mL for undiluted samples. However, these validation assays cannot be used to make inference regarding the ability of the assay to detect true changes, which is precisely the novelty of the data provided here.

More valid exposure measures have been sought that are not influenced by the choice of the self-report instrument used to collect data on smoking exposure [16] or on the more complex biochemical determination of exposure to tobacco through assessment of plasma levels of cotinine [17]. This is particularly important since it is known that tobacco exposure misclassification is differential, and higher rates of misstated tobacco use have been observed among certain groups of people, for example pregnant women [18], people with alcohol and depression history [19], or with cardiac and respiratory diseases [20,21]. A meta-analysis of clinical trials on smoking cessation demonstrated that the effect size was considerably influenced in five of the 21 studies included (24%) by the tobacco exposure measure used in the analyses, depending on the use of self-reported information or biochemical analysis of the exposure to tobacco [17]. A significant effect of the smoking cessation intervention was observed when self-reported information was used, but the association disappeared when analyses used biochemical results [17]. Interestingly, in two clinical smoking cessation studies carried out among pregnant women, a positive treatment effect was found when biochemical verification was used as the measure of exposure to tobacco, whereas no treatment effect was apparent when analysis was based on the self-reported information on smoking [19]. This difference may be explained by the desirability of the pregnant women to under-report their exposure to tobacco from the beginning of the study. In addition, the Hawthorne effect can also be responsible for the observed changes when people behave differently because they know they are being watched. It is thus clear that the use of the cotinine analysis may circumvent some of the biases that might be caused by e.g., the social undesirability of smoking or by memory bias, just as it may allow for assessment of some dimensions of duration and intensity of smoking, which may be difficult to capture through self-report [22]. In comparison with the nicotine half-life of 2 h, cotinine was demonstrated to have an extended half-life of 19 h [10], which allows cotinine levels to accumulate over the course of the day. Moreover, cotinine elimination is also slower than nicotine, which reflects in the fairly stable levels of cotinine over the day in comparison to oscillating levels of nicotine [19]. Therefore, cotinine stability over time and its potential to demonstrate long-term exposure to tobacco makes cotinine the ideal biomarker for determining tobacco exposure.

For various reasons, smoking assessment instruments have typically been using a rather simple two- or three-category question (e.g., smoker, non-smoker/never smoker, former smoker), and this clearly also fails to account for the many dimensions of the smoking habit [23]. Such simple categorization of smoking usually increases the influence of residual confounding and may lead to spurious findings [24]. As an example, it has been shown that an inverse relationship between body mass index and mortality could be ascribed to considerable residual confounding introduced by a very crude assessment of the smoking exposure information [25], a problem that has also been observed in studies of the relationship between periodontitis and systemic conditions [26].

Our calculations of the frequency of observed cotinine level reductions in a hypothetical smoking cessation study show the impact of measurement error on the possibility of making valid diagnosis of true changes in cotinine levels (i.e., with a false-positive error rate of 5% or less) as a function of the magnitude of the change. Our data indicate that the measurement error profile (Figure 2) of salivary cotinine level assessments allows for the valid detection of cotinine reductions in excess of 108 ng/mL (provided all study participants show such a reduction) and detection of cotinine reductions in excess of 200 ng/mL, provided that at least 28% of study participants show such a reduction. According to the present results, such reductions would correspond to 28% of a study group moving from heavy smoking to light smoking.

Even though the use of biochemical verification of cotinine may not capture the impact of smoking in a life course perspective, it does allow for the use of a continuous variable for smoking exposure, which is preferred from a statistical perspective, as it will reduce residual confounding by smoking [24,27]. It is worth highlighting that transforming a continuous variable, such as that representing cotinine level, into a two- or three-category variable for statistical purposes may also contribute to important residual confounding, especially in cases of a non-linear relationship between cotinine levels and the outcome (e.g., in studies of the effect of smoking on obesity, or on cardiovascular mortality) [24,28]. In addition, restricting the analyses to never smokers in an attempt to solve the problem of residual confounding due to smoking and avoid reverse causation, will clearly compromise the possibility of generalization of the findings. Therefore, it would be advisable to compare the results obtained based only on analysis of a never-smoking group with the results obtained from an analysis of the cotinine-adjusted data from the entire study population including smokers. Only then will it be possible to make inference regarding the possible generalizability of the results.

Determination of salivary cotinine levels may improve the quality and validity of the information on smoking behavior compared to self-reported information. The stability of cotinine in saliva may allow samples to be obtained in difficult-to-reach study areas, and reduce the costs of analyses.

## 4. Materials and Methods

This study was approved by the Central Denmark Region ethics committee (approval # 1-10-72-336-15). Eligible volunteers were current smokers aged between 18 and 70 years and were recruited using different means, including newspapers, flyers, social media, and website postings. All volunteers were invited to participate in a meeting during which they received oral and written information about the study. A total of 187 persons agreed to participate and were requested to fill a self-administered questionnaire to obtain information on their sociodemographic characteristics and the history of their smoking habit. The 172 participants who had provided self-reported information on their smoking habits were classified as Light (<10 cigarettes/day), Moderate (10–19 cigarettes/day) or Heavy (≥20 cigarettes/day) [29].

A single whole unstimulated saliva sample of 5 mL was collected from each participant by the passive drool method. Participants had been requested to avoid a large meal for 2 h prior to sampling, and they were asked to have abstained from alcohol for the past 12 h prior to sampling. Acidic or high sugar foods can compromise cotinine assay performance by lowering sample pH and influencing bacterial growth. Therefore, all samples were taken between 8 and 12 in the morning in order to aid the participants to cope with the avoidance of a meal prior to saliva collection. Before saliva collection, participants rinsed their mouths with tap water to remove food particles and ‘reset’ salivary secretion. To avoid sample dilution, participants then waited for 10 min before commencing sample collection. After saliva collection, the sample was frozen at −80 °C until all samples had been collected, which meant that samples were frozen at this temperature for a period up to 12 months.

### 4.1. Sample Processing and Cotinine Analyses

The study commenced when all samples had been collected. Samples were thawed; vortexed for 30 s and 100 μL of saliva was pipetted into each of six tubes. Each tube was allocated to a different storage condition: (I) Immediate cotinine level measurement (baseline assessment), (II) Regular mail posting at room temperature (PostNord, Aarhus, Denmark), (III) Storage at 4 °C for 30 days, (IV) Storage at 4 °C for 90 days, (V) Storage at −20 °C for 30 days, (VI) Storage at −20 °C for 90 days. Before analysis for cotinine, samples were brought to room temperature, vortexed for 15 s and then centrifuged at 1500× *g* for 15 min to remove particulate matter that could interfere with antibody binding before being diluted 1:10 using the assay kit dilution buffer. Cotinine levels were biochemically determined as the mean value of duplicate readings using a high sensitivity quantitative enzyme-linked immune-sorbent assay (ELISA) kit (Salimetrics, State College, PA, USA). The optical density was measured using a microplate reader at 450 nm (EL800, BioTek Instruments, Winooski, VT, USA) and the data were recorded after standard curve preparation.

In five samples the recorded cotinine levels were beyond the detection limits of the cotinine kit and data were not used in this study.

### 4.2. Statistical Analyses

The distributional characteristics of the cotinine contents of the samples for each of the six storage conditions were explored using the Shapiro-Francia test for normal data (proc sfrancia of Stata14.2; StataCorp, College Station, TX, USA), followed by a test for skewness and kurtosis (proc sktest of Stata14.2, StataCorp), to determine which distributional features would be the cause of non-normal distribution. Results showed that the hypothesis of normal distribution could be rejected for all but one storage condition (4 °C for 90 days), and that skewness in the data was the key reason (Tables S2 and S3). We therefore performed Box-Cox transformation (proc bcskew0 of StataSE 14.2, StataCorp) to remove skewness, but when comparing the resulting normal quantile plots (proc qnorm StataSE 14.2, StataCorp) of the data obtained after Box-Cox transformation with the normal quantile plots of the original data, the distributional improvements were only marginal (Figure S1). Therefore, in accordance with recommendations [30] and for ease of interpretation of the results, we decided to use the original data in all subsequent analyses. As a result, one can observe that the data spread and error were constant along the range of concentration on the reported units’ plot in Figure 1 [29]. This information can be seen as a slight fan shape in Figure 1 plots. In any case, for constant differences in concentration interval, the use of differences gives a more suitable portrayal of the difference between two measurements [31].

Description of the data was therefore based on measures of central tendency and dispersion. Agreement of the cotinine levels recorded when samples were analyzed immediately after thawing and after different storage conditions was assessed using Bland-Altman analyses [32]. The upper and lower limits of agreement (LoA) were calculated considering that approximately 95% of the differences between results would lie within 1.96 standard deviation [32]. In order to illustrate the differences in the measurements due to variation in the storage conditions, percentile differences were estimated [33].

In order to quantify the impact of the measurement errors on our ability to declare true change, we considered a hypothetical smoking cessation study in which salivary cotinine measurements were used to determine the outcome of a smoking cessation intervention. In such a study, the detection of change in cotinine levels is influenced by two error rates [34]: The type I error rate, P_Type I_, which is the proportion of participants with no real change that are measured as changed, and the false positive rate, P_f+_, which is the proportion of participants measured to have changed when no real change has occurred. If we denote the true, but unknown, proportion of participants with change P_True+_, and the observed proportion of participants measured as changed P_Change+_, we can quantify the false positive rate as follows:

P_f+_ = P_Type I_ × (1 − P_True+_)/P_Change+_

P_Type I_ can be estimated from reproducibility data, i.e., by measuring the samples twice or more, as has been done in the present study.

P_Change+_ is determined by the diagnostic rule set, i.e., ‘observed changes > X are considered diagnostic for real change’.

P_True+_ remains unknown, but the above formula may nonetheless be used to calculate an upper bound for P_f+_, assuming that P_True+_ is zero.

We sought to determine the minimum proportion of participants with a given observed cotinine level change (P_Change+_), which we could safely, i.e., with a P_f+_ of less than 5%, declare a real change that had not arisen as a result of error. We therefore used the above formula to calculate for each possible diagnostic threshold value, the proportion of participants (P_Change+_) that must show this or a larger change, when the measurement errors (P_Type I_), are distributed as shown by the differences observed here. In so doing, we pooled all differences between the immediate assessment of salivary cotinine level and the assessments made of each of the samples exposed to the five storage conditions.

Figure 2 outlines the relationship between the observed cotinine level change (the diagnostic threshold value) and the proportion of participants that should be observed with such or larger changes if the maximum false-positive rate is not to exceed 5%. As an example, suppose that 3% of duplicate measurements result in a cotinine difference ≥ 100 ng/mL and that a change of this magnitude has been observed in 65% of the participants in a hypothetical smoking cessation study, then application of the formula yields a maximum false positive rate of 4.62% (0.03/0.65), i.e., below the acceptable level of 5% [33]. The example therefore shows that if the frequency of observed changes ≥ 100 ng/mL is less than 65% or the error frequency is greater than the 3% assumed in the example, an unacceptably high portion of the observed changes will be false-positive changes.

## Figures and Tables

**Table 1:** Cotinine data description (in ng/mL) for the 182 samples treated according to the different storage protocols.

Storage Conditions	Mean ± SD	Median	Range
Immediate	456.6 ± 241.9	435.5	11.3–1318.0
Mail	446.4 ± 234.3	436.9	16.8–1208.0
4 °C for 30 days	467.7 ± 248.0	455.9	9.8–1209.0
4 °C for 90 days	438.8 ± 215.6	422.5	20.7–1023.0
−20 °C for 30 days	432.8 ± 230.3	411.2	9.4–1215.0
−20 °C for 90 days	443.9 ± 230.4	418.9	17.3–1166.0

**Table 2:** The mean value and standard errors of the salivary cotinine levels recorded immediately according to self-reported smoking exposure (*n* = 172).

Self-Reported Smoking Exposure	Mean (95% CI)	Standard Error
Light (<10 cigarettes per day)	346.4 (253.1–439.8)	45.7
Moderate (10–19 cigarettes per day)	416.0 (360.9–471.1)	27.6
Heavy (≥20 cigarettes per day)	542.0 (488.6–595.3)	26.8

## Supplementary Materials

The following are available online at https://www.mdpi.com/2218-1989/10/9/365/s1, Figure S1: Normal quantile plots of the original and Box-Cox transformed data demonstrating a marginal improvement in data distribution. The Box-Cox transformation value used in the analyses was 0.72433, Table S1: Bland-Altman analysis using the values from immediate analyses as the reference method, Table S2: Shapiro-Francia W´ test for normality, Table S3: Skewness and kurtosis tests for normality.
