# Supplementary material for: Effect of Sample Storage Conditions on Measurements of Salivary Cotinine Levels
Source: Metabolites. 2020 Sep 8;10(9):365. doi: 10.3390/metabo10090365 (PMC7569849; doi:10.3390/metabo10090365)
Supplement: Supplementary file 1 [file metabolites-10-00365-s001.pdf]

Table S1. Bland-Altman analysis using the values from immediate analyses as the reference method

Storage condition	Mean difference with immediate (95% CI)	Standard error	Width of limits of agreement
Mail	10.2 (-1.6;22.1)	6.0	156.8
4ºC for 30 days	-11.1 (-22.1;0.0)	5.6	148.1
4ºC for 90 days	17.6 (7.5;27.7)	5.1	135.4
-20ºC for 30 days	23.8 (14.1;33.6)	5.0	131.0
-20ºC for 90 days	13.4 (5.6;21.2)	3.9	104.0

Table S2. Shapiro-Francia W ´test for normality

Storage condition	W	V	Prob>z
Immediate	0.98	3.15	0.01
Mail	0.98	3.05	0.01
4ºC for 30 days	0.98	2.92	0.01
4ºC for 90 days	0.99	1.75	0.13
-20ºC for 30 days	0.98	3.49	0.01
-20ºC for 90 days	0.98	3.39	0.01

Table S3. Skewness and kurtosis tests for normality

Storage condition	Pr(Skewness)	Pr(Kurtosis)	Prob>chi2
Immediate	0.01	0.309	0.02
Mail	0.01	0.421	0.03
4ºC for 30 days	0.02	0.869	0.08
4ºC for 90 days	0.18	0.321	0.25
-20ºC for 30 days	0.01	0.213	0.01
-20ºC for 90 days	0.01	0.438	0.04

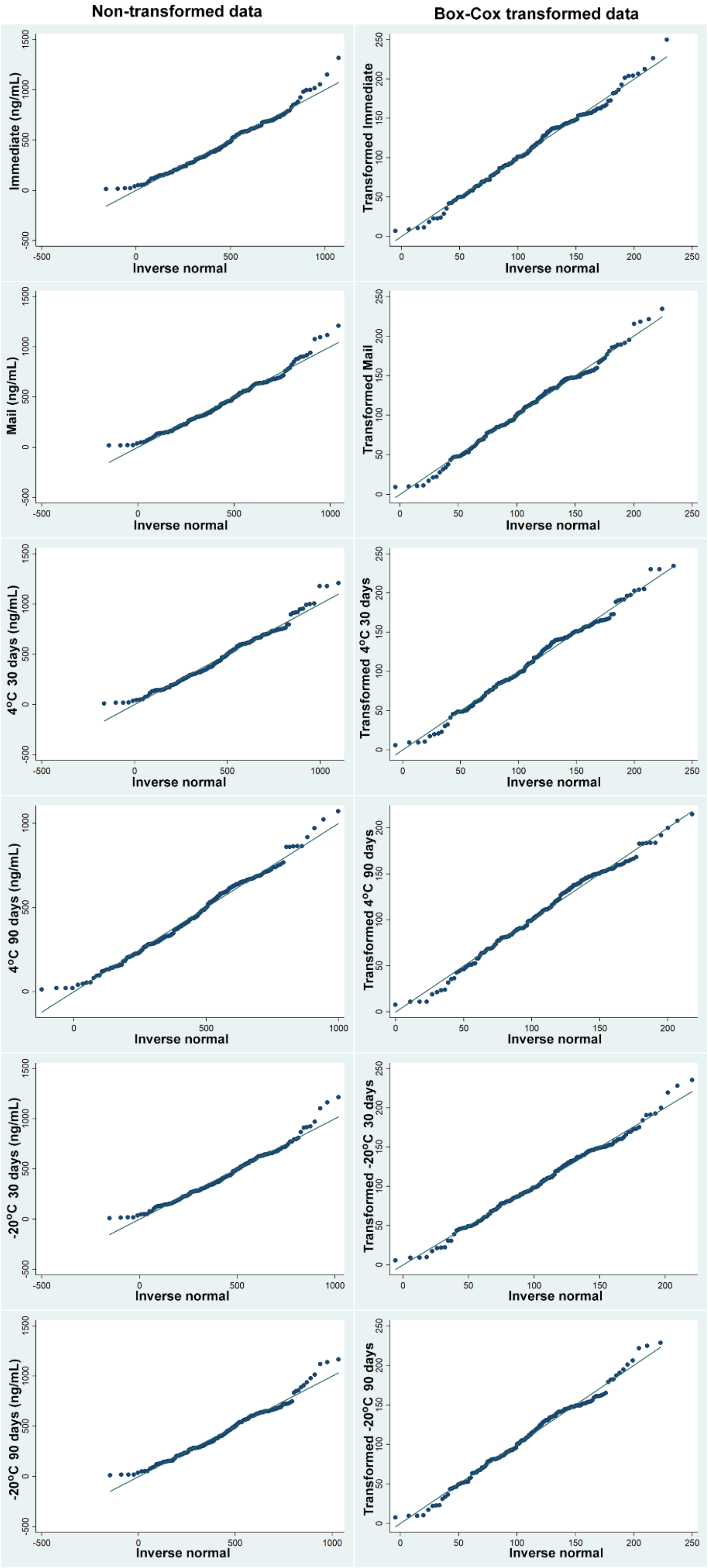

Figure S1. Normal quantile plots of the original and Box-Cox transformed data demonstrating a marginal improvement in data distribution. The Box-Cox transformation value used in the analyses was 0.72433.
